# Long-acting, progestin-based contraceptives and risk of breast, gynecological, and other cancers

**DOI:** 10.1093/jnci/djae282

**Published:** 2025-01-14

**Authors:** Karen M Tuesley, Katrina Spilsbury, Sallie-Anne Pearson, Peter Donovan, Andreas Obermair, Michael D Coory, Sitwat Ali, Nirmala Pandeya, Louise Stewart, Susan J Jordan, Penelope M Webb

**Affiliations:** School of Public Health, University of Queensland, Brisbane, QLD 4006, Australia; Population Health Program, QIMR Berghofer Medical Research Institute, Brisbane, QLD 4006, Australia; Institute for Health Research, The University of Notre Dame Australia, Fremantle, WA 6160, Australia; School of Population Health, University of New South Wales, Sydney, NSW 2052, Australia; Centre of Research Excellence in Medicines Intelligence, University of New South Wales, Sydney, NSW 2052, Australia; Clinical Pharmacology Department, Royal Brisbane and Women’s Hospital, Brisbane, QLD 4006, Australia; Faculty of Medicine, University of Queensland, Brisbane, QLD 4006, Australia; Queensland Centre for Gynaecological Cancer, Royal Brisbane and Women’s Hospital, Brisbane, QLD 4006, Australia; Centre for Clinical Research, University of Queensland, Brisbane, QLD 4006, Australia; Mater Research Institute, University of Queensland, Brisbane, QLD 4101, Australia; School of Public Health, University of Queensland, Brisbane, QLD 4006, Australia; Population Health Program, QIMR Berghofer Medical Research Institute, Brisbane, QLD 4006, Australia; School of Public Health, University of Queensland, Brisbane, QLD 4006, Australia; Population Health Program, QIMR Berghofer Medical Research Institute, Brisbane, QLD 4006, Australia; School of Population and Global Health, The University of Western Australia, Perth, WA 6009, Australia; School of Public Health, University of Queensland, Brisbane, QLD 4006, Australia; Population Health Program, QIMR Berghofer Medical Research Institute, Brisbane, QLD 4006, Australia; School of Public Health, University of Queensland, Brisbane, QLD 4006, Australia; Population Health Program, QIMR Berghofer Medical Research Institute, Brisbane, QLD 4006, Australia

## Abstract

**Background:**

Use of long-acting, reversible contraceptives has increased over the past 20 years, but an understanding of how they could influence cancer risk is limited.

**Methods:**

We conducted a nested case-control study among a national cohort of Australian women (n = 176 601 diagnosed with cancer between 2004 and 2013; 882 999 matched control individuals) to investigate the associations between the levonorgestrel intrauterine system, etonogestrel implants, depot-medroxyprogesterone acetate and cancer risk and compared these results with the oral contraceptive pill. We used conditional logistic regression to estimate odds ratios (OR) and 95% confidence intervals (CI).

**Results:**

Levonorgestrel intrauterine system and etonogestrel implant use was associated with breast cancer risk (OR = 1.26, 95% CI = 1.21 to 1.31, and OR = 1.24, 95% CI = 1.17 to 1.32, respectively), but depot-medroxyprogesterone acetate was not, except when used for 5 or more years (OR = 1.23, 95% CI = 0.95 to 1.59). Reduced risks were seen for levonorgestrel intrauterine system (≥1 years of use) in endometrial cancer (OR = 0.80, 95% CI = 0.65 to 0.99), ovarian cancer (OR = 0.71, 95% CI = 0.57 to 0.88), and cervical cancer (OR = 0.62, 95% CI = 0.51 to 0.75); for etonogestrel implant in endometrial cancer (OR = 0.21, 95% CI = 0.13 to 0.34) and ovarian cancer (OR = 0.76, 95% CI = 0.57 to 1.02); and for depot-medroxyprogesterone acetate in endometrial cancer (OR = 0.21, 95% CI = 0.13 to 0.34). Although levonorgestrel intrauterine system, etonogestrel implant and depot-medroxyprogesterone acetate were all associated with increased cancer risk overall, for etonogestrel implant, the risk returned to baseline after cessation, similar to the oral contraceptive pill. We were unable to adjust for all potential confounders, but sensitivity analyses suggested that adjusting for parity, smoking, and obesity would not have materially changed our findings.

**Conclusion:**

Long-acting, reversible contraceptives have similar cancer associations to the oral contraceptive pill (reduced endometrial and ovarian cancer risks and short-term increased breast cancer risk). This information may be helpful to women and their physicians when discussing contraception options.

## Introduction

Long-acting, reversible contraceptives, particularly progestin-based long-acting, reversible contraceptives, provide convenient, long-lasting, and highly effective female contraception.^1^ They include intrauterine devices, subdermal implants, and depot-medroxyprogesterone acetate injections, and their use has increased substantially over the past 20 years.^2-5^ Their advantages include long durations of action, ranging from a few months (injections) up to 7 years (intrauterine devices), and use when taking estrogen is contraindicated or undesirable.

Long-term combined oral contraceptive pill use substantially reduces endometrial and ovarian cancer risks,^6-9^ but current or recent users have slightly increased risks of breast and cervical cancers.^10-12^ As long-acting, reversible contraceptive use increases, it is important that we understand the potential effects on future cancer rates. There are currently few data on the effects of long-acting, reversible contraceptives on cancer risk, despite increasing use of these agents.

A 2023 meta-analysis reported 20% to 30% increased risks of breast cancer for premenopausal women who used progestogen-only contraceptives, regardless of type, that did not return to baseline until 10 or more years after last use.^13^ Two studies specifically investigating use of the levonorgestrel intrauterine system and ovarian cancer^14^^,^^15^ found a 40% to 50% reduction in risk. Similarly, a pooled analysis reported that use of depot-medroxyprogesterone acetate was associated with a 35% reduction in ovarian cancer risk.^16^ Studies of other cancers have been limited, although there are suggestions that the levonorgestrel intrauterine system may also protect against endometrial^17^ and cervical cancers.^18^

We investigated the associations between use of the levonorgestrel intrauterine system, 68-mg etonogestrel implants, and depot-medroxyprogesterone acetate and overall risk of developing cancer as well as risks of breast, endometrial, ovarian, cervical, and other individual cancers, using linked administrative health data. We compared our results with those for the oral contraceptive pill.

## Methods

### Study design and participants

We conducted a nested case-control study in a cohort of all Australian women (female sex assigned at birth) enrolled in Medicare, Australia’s universal health insurance scheme, on July 1, 2002.^19^ Women entered the cohort on the later of July 1, 2004 (yielding ≥2 years of dispensing history before cancer diagnosis) or their 20th birthday. Women younger than 55 years of age on July 1, 2002 (we were interested in premenopausal hormone use) with no prior cancer were eligible. Their records were linked to dispensing claims from the Pharmaceutical Benefits Scheme (PBS), the Australian Cancer Database, and the National Death Index (Figure S1).

### Case and control definition

**Figure 1.**
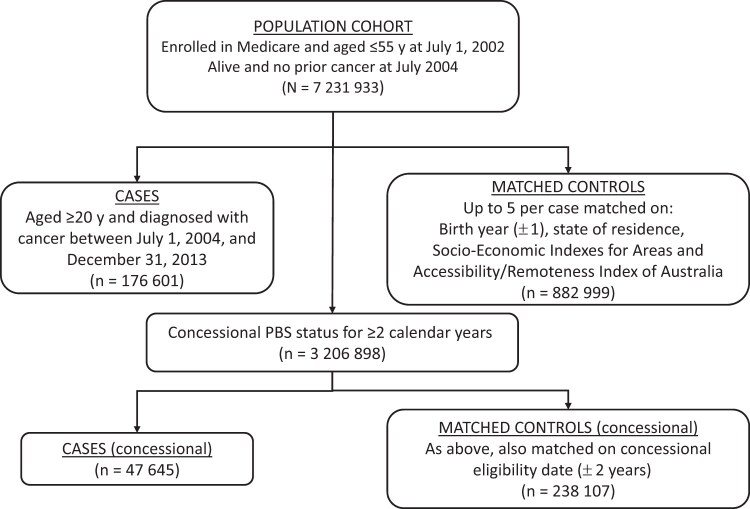
Flowchart of the selection of case and control individuals for the study. Abbreviation: PBS = Pharmaceutical Benefits Scheme.

In total, 176 601 women aged 20 years or older had a first cancer diagnosis between July 1, 2004, and December 31, 2013. Using the cancer diagnosis date as the index date, we matched up to 5 randomly selected control individuals to each case by birth year (±1), state of residence, Socio-Economic Indexes for Areas quintile, and remoteness category (Supplementary Methods).^20^^,^^21^ Women could be selected as a control for more than 1 case, and women with a cancer diagnosis could be selected as controls before their diagnosis. There were 882 999 matched control individuals (Figure 1).

### Concessional subcohort

We identified a subcohort of women who had been PBS concessional beneficiaries (who receive discounted medicines) for at least 2 years to assess depot-medroxyprogesterone acetate and oral contraceptive pill use because these low-cost medicines were not captured in the PBS for general beneficiaries before 2012 (see Supplementary Materials for details). Women diagnosed with cancer while eligible for the concessional subcohort (n = 47 645) were individually matched to a maximum of 5 control individuals (n = 238 107) who were also eligible for the subcohort at the index date, using the same criteria as noted plus concessional eligibility date (±2 years) (Figure 1).

### Exposure variables

We classified women as levonorgestrel intrauterine system, etonogestrel implant, or depot-medroxyprogesterone acetate users (Table S3) at the date of their first dispensing record. Levonorgestrel implants are not available in Australia. We classified women as oral contraceptive pill users at the date of their second dispensing record. Etonogestrel implant and levonorgestrel intrauterine system have been available in Australia only since 2001 and 2002, respectively, so PBS records capture most use; however, we will have misclassified women who only used depot-medroxyprogesterone acetate or oral contraceptive pills before July 2002 as nonusers. We estimated duration of levonorgestrel intrauterine system and etonogestrel implant use as the period from date of first supply until the earlier of the index date or 5 or 3 years (based on clinical guidelines)^22^^,^^23^ after the last dispensing, respectively, recognizing that this would overestimate duration if devices were removed earlier (studies suggest discontinuation rates of 15% to 25% at 1 year^24^^,^^25^). We similarly estimated duration of depot-medroxyprogesterone acetate and oral contraceptive pill use from first use until the earlier of the index date or 3 months after the last dispensing, recognizing that we would underestimate duration of use before July 2002.

We assigned categories of use by combining duration and recency. Women were defined as recent users if their estimated end of use date was within 2 years of their index date and as former users when it was more than 2 years before the index date. We then categorized recent users as short term (<12 months), medium term (1 to <5 years), and long term (≥5 years). Because we assumed that levonorgestrel intrauterine systems/etonogestrel implants were removed after 5 and 3 years, respectively, women classified as former levonorgestrel intrauterine system/etonogestrel implant users had at least 5 or 3 years of use, respectively. For depot-medroxyprogesterone acetate and oral contraceptive pills, we categorized them separately as short-term former use (<12 months).

### Covariates

We used PBS records to define women as users of menopausal hormone therapy if, before their index date, they were dispensed hormone therapy at least twice within an 18-month period. To adjust for potential confounding by comorbidity, we used the weighted Rx-Risk Comorbidity Score^26^ (Table S5).

### Statistical analyses

We used conditional logistic regression to estimate odds ratios (ORs) and 95% confidence intervals (CI) for the associations between long-acting, reversible contraceptives use and the risk of cancer overall and by type: breast, endometrial, cervical, and epithelial ovarian cancers plus colorectal, lung, and thyroid cancers and melanoma for secondary analyses (Table S1); all other cancers were grouped as “other” (Table S2). We initially assessed associations for the levonorgestrel intrauterine system and etonogestrel implants separately in the full cohort, and then included both in the same model. As the levonorgestrel intrauterine system is often used to treat heavy menstrual bleeding,^27^ a potential symptom of gynecological cancer, we additionally assessed the associations with use for more than 1 year. All models were adjusted for the weighted Rx-Risk Comorbidity Score.

We repeated these models in the concessional subcohort, where we also evaluated depot-medroxyprogesterone acetate and oral contraceptive pills and assessed the effects of adjusting for use of these contraceptives and of menopausal hormone therapy. To evaluate whether the overall risk-benefit profile of long-acting, reversible contraceptives varied with age, we conducted additional analyses of cancer risk overall, stratified by age at diagnosis in 10-year groups. Analyses were performed in SAS, version 9.4, statistical software (SAS Institute Inc).

### Sensitivity analyses

We did not have information about potential confounders such as obesity, smoking, and parity for the national dataset; therefore, we conducted sensitivity analyses to assess the likely effects on our results. We first restricted analyses to women in Western Australia, where we had additional hospital morbidity information (99% of case individuals and 98% of control individuals had a hospital record) and could exclude those individuals who had undergone hysterectomy or oophorectomy (where necessary) and additionally adjusted for smoking and parity (Table S4). We then used PBS data to predict obesity^28^ to assess confounding in the concessional subcohort (Supplementary Methods). We used quantitative bias analysis^29^ to quantify the potential bias for underreporting of smoking status in hospital records.

Because we did not have information about depot-medroxyprogesterone acetate or oral contraceptive pill use before July 2002, we also conducted sensitivity analyses for breast cancer, first excluding women aged 45 to 54 years on July 1, 2002, because they would be most likely to be former contraceptive users and second, commencing follow-up in 2008 to ensure that we had a minimum of 6 years of prescribing history for all women. In a final sensitivity analysis, we compared exclusive use of each contraceptive with no use of any long-acting, reversible contraceptives or oral contraceptive pill as the reference group.

## Results

Characteristics of the full cohort are presented in Table S6. As we excluded women older than 55 years of age in 2002, the oldest women in the study were 67 years of age. Most cancers were diagnosed between the ages of 50 and 59 years (43%) or 40 and 49 years (27%), with only 4% of women diagnosed before they were 30 years of age. Compared with the full cohort, women with cancer in the concessional subcohort (Table S7) tended to live in more disadvantaged areas and be older.

Results for the concessional subcohort for levonorgestrel intrauterine system and etonogestrel implant use were generally consistent with the full cohort, so we focused on the results for the levonorgestrel intrauterine system and etonogestrel implants from the full cohort and depot-medroxyprogesterone acetate from the concessional subcohort.

### All cancer

**Figure 2.**
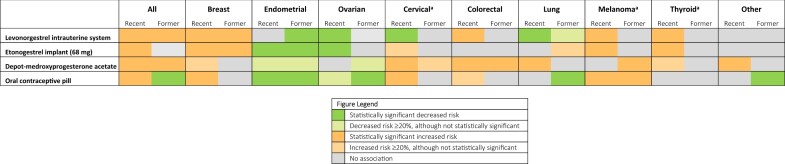
Summary of associations between recent and former contraceptive use and risk of cancer. ^a^ Possible uncontrolled confounding or detection bias.

Overall, levonorgestrel intrauterine system use was associated with a 25% increased risk of being diagnosed with cancer (Table 1, Figure 2). This result was partly because of the strong association seen for recent short-term use (OR = 1.69), a likely artefact (see below). When we excluded users of less than 1 year, the association attenuated (OR = 1.16, 95% CI = 1.13 to 1.19). Recent medium-term and long-term use was associated with a 14% to 18% increased risk, with an 11% increase seen for former users (with median time since last use of 3 years).

Any use of the etonogestrel implant was associated with a 9% increased cancer risk, but this increase was restricted to recent use, regardless of duration (eg, long-term use: OR = 1.17, 95% CI = 1.07 to 1.28), and there was no association with former use. Any depot-medroxyprogesterone acetate use was associated with an 11% increased risk of cancer, with higher risks for individuals with longer-term use (recent long-term use: OR = 1.28, 95% CI = 1.12 to 1.47). The increased risk persisted for former users with more than 12 months of use. Similarly, recent long-term oral contraceptive pill use (≥5 years) was associated with a 27% increase in cancer risk, but former users had no increased risk. There was no consistent variation by age (Table S8).

### Breast cancer

Ever use of levonorgestrel intrauterine system or etonogestrel implant was associated with a 26% and 24% increased risk of breast cancer, respectively, compared with no use (Table 1). In the full study population, the risk associated with levonorgestrel intrauterine system was higher for longer duration of use and persisted among former users who, by definition, had at least 5 years of use but at least 2 years of nonuse. Recent etonogestrel implant use was associated with a 30% to 40% increased risk, regardless of duration. The risk was lower but still statistically significantly elevated 2 or more years since stopping (OR = 1.12, 95% CI = 1.02 to 1.23).

**Table 1.** Association between long-acting, progestin-based contraceptives and all, breast, endometrial, epithelial ovarian, and cervical cancers

	All cancer			Breast cancer			Endometrial cancer			Epithelial ovarian cancer			Cervical cancer		
Medication used^a^	Cases	Controls	OR (95% CI)^b^	Cases	Controls	OR (95% CI)^b^	Cases	Controls	OR (95% CI)^b^	Cases	Controls	OR (95% CI)^b^	Cases	Controls	OR (95% CI)^b^
Full cohort, No.	176 601	882 999		67 470	337 347		7290	36 450		4752	23 760		4755	23 775	
Levonorgestrel intrauterine system, %															
No use	95.5	96.4	(Referent)	95.2	96.2	(Referent)	92.3	97.5	(Referent)	97.1	96.8	(Referent)	95.7	95.3	(Referent)
Any use	4.5	3.6	1.25 (1.21 to 1.28)	4.8	3.8	1.26 (1.21 to 1.31)	7.7	2.5	3.58 (3.19 to 4.02)	2.9	3.2	0.90 (0.75 to 1.08)	4.3	4.7	0.89 (0.77 to 1.04)
Any use (≥1 y)	3.5	3.0	1.16 (1.13 to 1.19)	4.1	3.2	1.28 (1.22 to 1.34)	1.5	2.1	0.80 (0.65 to 0.99)	2.0	2.8	0.71 (0.57 to 0.88)	2.4	3.8	0.62 (0.51 to 0.75)
Use category and duration															
Recent: short term	1.0	0.6	1.69 (1.60 to 1.79)	0.7	0.6	1.16 (1.04 to 1.28)	6.2	0.4	21.2 (17.1 to 26.3)	0.9	0.4	2.31 (1.61 to 3.33)	1.9	0.9	2.11 (1.63 to 2.71)
Recent: medium-term	2.3	2.0	1.18 (1.14 to 1.22)	2.6	2.1	1.27 (1.20 to 1.34)	1.0	1.3	0.92 (0.72 to 1.18)	1.4	1.8	0.76 (0.58 to 0.98)	1.8	2.7	0.65 (0.52 to 0.82)
Recent: long term	0.9	0.8	1.14 (1.08 to 1.21)	1.1	0.9	1.33 (1.22 to 1.44)	0.4	0.5	0.82 (0.54 to 1.23)	0.4	0.8	0.53 (0.34 to 0.83)	0.4	0.9	0.46 (0.29 to 0.73)
Former^c^	0.3	0.3	1.11 (1.01 to 1.22)	0.4	0.3	1.26 (1.10 to 1.44)	0.1	0.3	0.30 (0.13 to 0.68)	0.2	0.2	0.96 (0.48 to 1.90)	0.2	0.2	0.86 (0.42 to 1.77)
Etonogestrel implant, %															
No use	97.6	97.8	(Referent)	97.8	98.2	(Referent)	99.8	99	(Referent)	98.9	98.5	(Referent)	94.7	95.2	(Referent)
Any use	2.4	2.2	1.09 (1.06 to 1.13)	2.2	1.8	1.24 (1.17 to 1.32)	0.2	1.0	0.21 (0.13 to 0.34)	1.1	1.5	0.76 (0.57 to 1.02)	5.3	4.8	1.12 (0.97 to 1.29)
Use category and duration															
Recent: short term	0.2	0.2	1.17 (1.05 to 1.31)	0.2	0.1	1.32 (1.07 to 1.63)	—	0.1	—^d^	<0.1	<0.2	0.81 (0.24 to 2.78)	0.6	0.4	1.25 (0.82 to 1.92)
Recent: medium term	1.0	0.9	1.11 (1.05 to 1.17)	0.9	0.7	1.30 (1.18 to 1.42)	<0.1	<0.5	0.13 (0.05 to 0.36)	<0.5	<0.8	0.63 (0.40 to 0.98)	2.2	2.2	1.00 (0.80 to 1.24)
Recent: long term	0.4	0.3	1.17 (1.07 to 1.28)	0.4	0.3	1.40 (1.22 to 1.61)	<0.1	<0.3	0.14 (0.03 to 0.64)	<0.2	<0.3	0.82 (0.39 to 1.74)	0.8	0.6	1.37 (0.95 to 1.96)
Former^c^	0.8	0.8	1.03 (0.97 to 1.09)	0.8	0.7	1.12 (1.02 to 1.23)	0.2	0.4	0.29 (0.15 to 0.58)	0.4	0.5	0.94 (0.58 to 1.52)	1.7	1.6	1.14 (0.89 to 1.46)
Concessional subcohort, No.	47 645	238 107		15 391	76 912		2206	11 024		1273	6365		1702	8510	
Depot-medroxyprogesterone acetate, %															
No use	96.2	96.6	(Referent)	97	97	(Referent	99	98.3	(Referent)	97.9	97.4	(Referent)	90.1	92.6	(Referent)
Any use	3.8^e^	3.4^e^	1.11 (1.05 to 1.17)	3.1	3.0	1.01 (0.91 to 1.12)	1.0	1.7	0.52 (0.32 to 0.84)	2.1	2.6	0.82 (0.54 to 1.25)	9.9	7.4	1.38 (1.15 to 1.67)
Use category and duration															
Recent: short term	0.6	0.5	1.10 (0.96 to 1.26)	0.3	0.4	0.89 (0.66 to 1.19)	0.4	0.2	1.61 (0.67 to 3.88)	<0.5	<0.5	0.76 (0.22 to 2.67)	1.8	1.1	1.59 (1.05 to 2.43)
Recent: medium term	0.7	0.6	1.16 (1.03 to 1.31)	0.5	0.5	1.00 (0.78 to 1.28)	<0.3	<0.3	0.54 (0.16 to 1.85)	0.8	0.5	1.53 (0.74 to 3.15)	2.1	1.6	1.33 (0.91 to 1.95)
Recent: long term	0.6	0.5	1.28 (1.12 to 1.47)	0.5	0.4	1.23 (0.95 to 1.59)	<0.3	<0.3	0.25 (0.03 to 1.87)	<0.5	<0.5	0.71 (0.20 to 2.50)	1.5	0.9	1.74 (1.09 to 2.78)
Former: short term	1.2	1.2	1.02 (0.93 to 1.12)	1.1	1.1	0.98 (0.83 to 1.16)	0.3	0.6	0.66 (0.29 to 1.51)	0.5	1.0	0.54 (0.23 to 1.26)	2.8	2.5	1.20 (0.87 to 1.66)
Former: ≥1 y^c^	0.8	0.7	1.13 (1.01 to 1.27)	0.7	0.6	1.08 (0.87 to 1.34)	<0.3	<0.5	0.26 (0.06 to 1.11)	<0.5	<0.5	0.80 (0.31 to 2.07)	1.7	1.3	1.33 (0.87 to 2.03)
Oral contraceptive pill															
No use	86.8	87.3	(Referent)	86.5	88.5	(Referent)	96.6	93.4	(Referent)	92.2	89.9	(Referent)	73.4	74.7	(Referent)
Any use	13.2^e^	12.7^e^	1.06 (1.02 to 1.09)	13.5	11.5	1.24 (1.17 to 1.31)	3.4	6.6	0.44 (0.34 to 0.57)	7.8	10.2	0.72 (0.56 to 0.91)	26.6	25.3	1.09 (0.96 to 1.23)
Use category and duration															
Recent: short-term	1.2	1.0	1.14 (1.03 to 1.25)	1.0	0.8	1.26 (1.05 to 1.51)	0.6	0.4	1.02 (0.49 to 2.11)	0.8	0.7	1.02 (0.50 to 2.07)	2.5	2.0	1.25 (0.87 to 1.78)
Recent: medium term	3.6	3.1	1.19 (1.13 to 1.26)	3.9	2.7	1.51 (1.37 to 1.67)	0.6	1.4	0.30 (0.16 to 0.56)	2.0	2.6	0.76 (0.49 to 1.18)	7.0	6.0	1.21 (0.97 to 1.50)
Recent: long-term	2.3	1.9	1.27 (1.18 to 1.36)	2.7	1.7	1.70 (1.51 to 1.92)	0.3	0.8	0.24 (0.10 to 0.60)	1.4	1.4	0.89 (0.52 to 1.53)	6.6	4.4	1.51 (1.19 to 1.91)
Former: short-term	2.8	3.0	0.94 (0.88 to 1.00)	2.6	2.8	0.98 (0.88 to 1.10)	1.1	1.7	0.53 (0.33 to 0.85)	2.0	2.5	0.76 (0.49 to 1.18)	4.5	5.8	0.82 (0.63 to 1.05)
Former: ≥1 y^c^	3.4	3.7	0.93 (0.88 to 0.98)	3.4	3.5	1.04 (0.94 to 1.14)	0.8	2.3	0.33 (0.20 to 0.55)	1.6	3.0	0.51 (0.31 to 0.82)	6.0	7.1	0.89 (0.71 to 1.12)

Abbreviation: OR = odds ratio.

a Short-term use: <12 mo; medium-term use: 1 to <5 y; long-term use: ≥5 y.

b All models were adjusted for weighted Rx-Risk score at index date; matched by age, Socio-Economic Indexes for Areas, remoteness, and registered state; and included other contraceptives in the same model. Models using the concessional subcohort additionally adjusted for menopausal hormone therapy use.

c For former users (≥1 y for depot-medroxyprogesterone acetate and oral contraceptive pill), median (IQR) time in years since last use, levonorgestrel intrauterine system: 3.0 (2.5-3.8); etonogestrel implant: 4.3 (3.0-5.8); depot-medroxyprogesterone acetate: 4.2 (3.0-6.0); oral contraceptive pill: 4.2 (3.0-5.9).

d No cases in this category.

e Our data includes medicine use only from 2002; therefore, we will have misclassified women who only used depot-medroxyprogesterone acetate or the oral contraceptive pill before July 2002 as nonusers; therefore, women who used it may be underascertained.

In contrast, former depot-medroxyprogesterone acetate use or recent short-term and medium-term use was not associated with risk of breast cancer, but there was the suggestion of an association for recent long-term use (OR = 1.23, 95% CI = 0.95 to 1.59). The results were essentially unaltered when we restricted the cohort to women younger than 45 years of age on July 1, 2002, or women diagnosed after 2008, suggesting that they were not biased by the lack of information about depot-medroxyprogesterone acetate use before 2002 (Table S9). Our sensitivity analysis among women in Western Australia showed that adjustment for smoking attenuated the overall estimate toward the null (Table S10).

We saw the expected associations for the oral contraceptive pill with recent long-term use associated with a 70% increased risk that returned to baseline for women who had stopped use for at least 2 years. Sensitivity analyses did not materially change these results (Tables S9 and S10).

### Endometrial cancer

Women who commenced levonorgestrel intrauterine system use within 12 months of the index date had a high risk of being diagnosed with endometrial cancer compared with never users (OR = 21.2, 95% CI = 17.1 to 26.3) (Table 1), likely reflecting use for treatment during work-up for endometrial cancer rather than a causal effect. Use of more than 1 year was associated with reduced risk (OR = 0.80, 95% CI = 0.65 to 0.99), with a 70% reduced risk for former users (7 years since last dispensing) (OR = 0.30, 95% CI = 0.13 to 0.68).

Etonogestrel implant and depot-medroxyprogesterone acetate use was associated with substantially reduced risks of endometrial cancer compared with never use (OR = 0.21, 95% CI = 0.13 to 0.34, and OR = 0.52, 95% CI = 0.32 to 0.84, respectively) that persisted among former users. These associations were similar to those seen for the oral contraceptive pill.

### Epithelial ovarian cancer

As for endometrial cancer, women who commenced levonorgestrel intrauterine system use within 12 months of the index date had an increased risk of epithelial ovarian cancer compared with never users (OR = 2.31, 95% CI = 1.61 to 3.33), but long-term use was associated with a 47% reduced risk (Table 1). Recent use of the etonogestrel implant was also associated with lower risk (eg, medium-term use: OR = 0.63, 95% CI = 0.40 to 0.98). For both the levonorgestrel intrauterine system and the etonogestrel implant, risk returned close to baseline among former users. We did not find any clear associations with depot-medroxyprogesterone acetate, but most risk estimates were below 1.0 (Table 2). Oral contraceptive pill use was associated with a reduced risk that persisted after cessation.

**Table 2.** Association between long-acting, progestin-based contraceptives and colorectal, lung, melanoma, thyroid, and other cancers.

	Colorectal cancer			Lung cancer			Melanoma			Thyroid cancer			Other cancers		
Medication used^a^	Cases	Controls	OR (95% CI)^b^	Cases	Controls	OR (95% CI)^b^	Cases	Controls	OR (95% CI)^b^	Cases	Controls	OR (95% CI)^b^	Cases	Controls	OR (95% CI)^b^
Full cohort, No.	14 558	72 790		8505	42 521		23 171	115 853		9578	47 890		36 523	182 614	
Levonorgestrel intrauterine system, %															
No use	96.4	96.9	(Referent)	98.1	97.5	(Referent)	95.0	95.9	(Referent)	93.9	95.5	(Referent)	96.3	96.7	(Referent)
Any use	3.6	3.1	1.17 (1.06 to 1.29)	1.9	2.5	0.73 (0.61 to 0.86)	5.0	4.1	1.23 (1.15 to 1.32)	6.1	4.5	1.38 (1.25 to 1.52)	3.7	3.3	1.11 (1.04 to 1.18)
Use category and duration															
Recent: short term	0.6	0.5	1.16 (0.92 to 1.48)	0.3	0.3	0.74 (0.46 to 1.16)	0.9	0.7	1.20 (1.03 to 1.40)	1.1	0.8	1.30 (1.04 to 1.61)	0.8	0.5	1.44 (1.26 to 1.64)
Recent: medium term	2.0	1.6	1.24 (1.09 to 1.42)	1.0	1.3	0.74 (0.59 to 0.94)	2.9	2.2	1.31 (1.20 to 1.43)	3.3	2.4	1.45 (1.28 to 1.65)	1.9	1.8	1.03 (0.95 to 1.12)
Recent: long-term	0.7	0.7	1.05 (0.85 to 1.31)	0.5	0.6	0.74 (0.53 to 1.04)	1.0	0.9	1.15 (0.99 to 1.33)	1.3	1.0	1.38 (1.13 to 1.68)	0.7	0.7	1.02 (0.89 to 1.17)
Former	0.3	0.3	1.11 (0.81 to 1.52)	0.2	0.3	0.58 (0.33 to 1.01)	0.3	0.3	1.07 (0.83 to 1.38)	0.4	0.3	1.09 (0.75 to 1.59)	0.3	0.3	1.18 (0.97 to 1.45)
Etonogestrel implant, %															
No use	98.2	98.2	(Referent)	99.0	99.0	(Referent)	96.5	96.7	(Referent)	96.1	96.5	(Referent)	97.7	97.8	(Referent)
Any use	1.8	1.8	1.04 (0.91 to 1.19)	1.0	1.0	0.97 (0.76 to 1.25)	3.5	3.3	1.07 (0.99 to 1.16)	3.9	3.5	1.09 (0.97 to 1.22)	2.3	2.2	1.04 (0.97 to 1.13)
Use category and duration															
Recent: short term	0.1	0.2	0.72 (0.44 to 1.18)	0.1	0.1	1.46 (0.59 to 3.63)	0.4	0.3	1.27 (1.00 to 1.62)	0.4	0.3	1.47 (1.04 to 2.07)	0.2	0.2	1.02 (0.80 to 1.31)
Recent: medium term	0.8	0.7	1.16 (0.94 to 1.42)	0.3	0.4	0.84 (0.56 to 1.26)	1.5	1.5	1.03 (0.92 to 1.16)	1.8	1.5	1.21 (1.02 to 1.43)	1.0	0.9	1.08 (0.96 to 1.21)
Recent: long term	0.3	0.2	1.34 (0.97 to 1.86)	0.1	0.2	0.47 (0.22 to 1.02)	0.5	0.5	1.17 (0.96 to 1.43)	0.4	0.5	0.81 (0.58 to 1.14)	0.4	0.3	1.09 (0.90 to 1.32)
Former	0.7	0.6	0.89 (0.70 to 1.12)	0.5	0.4	1.31 (0.91 to 1.87)	1.1	1.1	1.02 (0.89 to 1.17)	1.2	1.2	0.96 (0.78 to 1.17)	0.7	0.7	0.99 (0.86 to 1.13)
Concessional subcohort	4124	20 618		3525	17 617		5346	26 713		2454	12 260		11 412	57 046	
Depot-medroxyprogesterone acetate, %															
No use	96.9	97.7	(Referent)	97.2	98.0	(Referent)	94.6	94.8	(Referent)	94.4	94.9	(Referent)	95.8	96.5	(Referent)
Any use	3.1	2.3	1.34 (1.09 to 1.65)	2.8	2.0	1.36 (1.08 to 1.73)	5.4	5.2	1.04 (0.91 to 1.19)	5.6	5.1	1.06 (0.87 to 1.29)	4.2	3.5	1.20 (1.08 to 1.33)
Use category and duration															
Recent: short term	0.3	0.3	0.92 (0.48 to 1.76)	0.3	0.2	1.98 (0.99 to 3.96)	0.8	0.9	0.89 (0.64 to 1.24)	0.9	0.9	1.01 (0.64 to 1.59)	0.7	0.5	1.34 (1.04 to 1.73)
Recent: medium term	0.5	0.4	1.07 (0.65 to 1.76)	0.3	0.3	1.04 (0.55 to 1.97)	1.2	1.1	1.17 (0.89 to 1.54)	1.1	0.9	1.25 (0.82 to 1.92)	0.9	0.7	1.33 (1.06 to 1.65)
Recent: long-term	0.5	0.3	1.75 (1.07 to 2.86)	0.6	0.3	1.63 (0.96 to 2.76)	0.6	0.6	1.04 (0.72 to 1.52)	0.8	0.7	1.18 (0.71 to 1.96)	0.6	0.4	1.41 (1.08 to 1.84)
Former: short term	0.9	0.5	1.18 (0.82 to 1.69)	0.9	0.7	1.39 (0.93 to 2.08)	1.6	1.7	0.90 (0.71 to 1.15)	1.9	1.7	1.07 (0.77 to 1.49)	0.7	0.6	1.08 (0.90 to 1.29)
Former: ≥1 y	0.9	0.8	1.98 (1.33 to 2.94)	0.6	0.5	1.11 (0.67 to 1.80)	1.2	0.9	1.34 (1.01 to 1.78)	0.9	1.0	0.83 (0.52 to 1.34)	1.3	1.2	1.04 (0.81 to 1.33)
Oral contraceptive pill, %															
No use	90.3	90.5	(Referent)	93.8	92.4	(Referent)	78.4	81.5	(Referent)	81.3	81.0	(Referent)	88.2	87.4	(Referent)
Any use	9.7	9.5	1.04 (0.92 to 1.18)	6.2	7.6	0.80 (0.68 to 0.93)	21.6	18.5	1.27 (1.17 to 1.37)	18.7	19.0	0.97 (0.86 to 1.10)	11.8	12.6	0.91 (0.85 to 0.98)
Use category and duration															
Recent: short term	0.6	0.8	0.81 (0.53 to 1.23)	0.6	0.4	1.33 (0.80 to 2.21)	2.2	1.9	1.24 (1.00 to 1.53)	1.8	1.7	1.07 (0.77 to 1.49)	1.1	1.1	1.03 (0.84 to 1.25)
Recent: medium term	2.6	2.1	1.22 (0.98 to 1.53)	1.6	1.6	1.00 (0.74 to 1.36)	5.9	5.0	1.29 (1.13 to 1.47)	5.1	4.7	1.09 (0.88 to 1.34)	3.1	3.2	0.98 (0.87 to 1.11)
Recent: long term	1.6	1.4	1.17 (0.88 to 1.55)	0.8	1.0	0.77 (0.51 to 1.16)	3.3	2.6	1.39 (1.17 to 1.66)	2.7	2.9	0.93 (0.71 to 1.23)	1.9	1.8	1.01 (0.87 to 1.18)
Former: short term	2.2	2.2	1.05 (0.83 to 1.32)	1.2	2.0	0.63 (0.46 to 0.86)	4.7	4.2	1.22 (1.05 to 1.41)	4.3	4.6	0.93 (0.75 to 1.15)	2.6	2.9	0.87 (0.76 to 0.99)
Former: ≥1 y	2.7	3.0	0.93 (0.75 to 1.15)	1.9	2.7	0.74 (0.57 to 0.96)	5.5	4.8	1.26 (1.10 to 1.44)	4.7	5.1	0.91 (0.73 to 1.12)	3.0	3.6	0.82 (0.73 to 0.92)

Abbreviation: OR = odds ratio.

a Short-term use: <12 mo; medium-term use: 1 to <5 y; long-term use: ≥5 y.

b All models were adjusted for weighted Rx-Risk score at index date; matched by age, Socio-Economic Indexes for Areas, remoteness, and registered state; and included other contraceptives in the same model. Models using the concessional subcohort were additionally adjusted for menopausal hormone therapy use.

### Cervical cancer

Recent commencement of levonorgestrel intrauterine system use was also associated with an increased risk of cervical cancer (OR = 2.11, 95% CI = 1.63 to 2.71) (Table 1). After 12 months of use, however, the risk was reduced (OR = 0.62, 95% CI = 0.51 to 0.75). For the etonogestrel implant, ever use was associated with a small but not statistically significant increased risk (OR = 1.12, 95% CI = 0.97 to 1.29), with a stronger association seen for depot-medroxyprogesterone acetate (OR = 1.38, 95% CI = 1.15 to 1.67). The sensitivity analysis among women in Western Australia showed that adjustment for smoking attenuated the estimate by 0.05 for the etonogestrel implant and 0.1 to 0.2 for depot-medroxyprogesterone acetate. Recent long-term but not former oral contraceptive pill use was also associated with an increased risk.

### Other cancers

To understand the full potential effects of long-acting, reversible contraceptives, we also evaluated the associations for other common cancers (Table 2), with the results for the full cohort shown for the levonorgestrel intrauterine system and the etonogestrel implant and for the concessional subcohort for depot-medroxyprogesterone acetate and the oral contraceptive pill. Ever use of the levonorgestrel intrauterine system was associated with an increased risk of colorectal cancer, melanoma, thyroid cancer, and all other noncategorized cancers combined but a reduced risk of lung cancer.

Overall, use of the etonogestrel implant was not associated with other cancers, although recent short-term use was associated with an increased risk of melanoma, and recent short-term and medium-term use was associated with increased thyroid cancer risk. Use of depot-medroxyprogesterone acetate was associated with statistically significantly increased risks of colorectal cancer, lung cancer, and all other cancers combined. Our sensitivity analysis using women in Western Australia showed an attenuation toward the null for lung cancer when we adjusted for smoking (Table S10). Use of the oral contraceptive pill was associated with an increased risk of melanoma and a reduced risk of lung cancer and all other cancers combined, particularly for former users.

### Other sensitivity analyses

Unless otherwise noted above, our sensitivity analyses adjusting for smoking, parity, and hysterectomy and oophorectomy and for predicted obesity did not materially change the results (Tables S10 and S11). For the Western Australia cohort, 35% had smoking recorded in a hospital record (33% for controls only). The proportion of Australian adult women who ever smoked ranged from 45% in 2002 to 37% in 2013.^30^ Quantitative bias analysis assuming a 20% underreporting of smoking and the known associations between smoking and each cancer did not alter the smoking-adjusted estimates by more than 0.01. Our sensitivity analysis with no long-acting, reversible contraceptive/oral contraceptive pill as the reference (Table S12) produced estimates consistent with the concessional subcohort analysis results for breast cancer (Table S9).

## Discussion

Our study shows that all long-acting, reversible contraceptives, particularly recent use, are associated with overall increased cancer risk that is of similar or greater magnitude to the oral contraceptive pill. These associations were primarily driven by the increased breast cancer risks among recent users, with some attenuation from the reduced risks observed for gynecological cancers. Associations differed between the types of long-acting, reversible contraceptives for specific cancers and when duration and timing of use were considered.

Recent levonorgestrel intrauterine system and etonogestrel implant use was associated with 20% to 40% increased breast cancer risk, and use of 12 months or longer was associated with decreased endometrial and epithelial ovarian cancer risk. The high risk of endometrial cancer for women with less than 12 months of levonorgestrel intrauterine system use probably reflects the use of the levonorgestrel intrauterine system to treat hyperplasia and symptoms of early-stage endometrial cancer.^27^^,^^31^ Similar phenomena may also explain the more modest short-term increases in ovarian and cervical cancer risk; for example, cervical cancer screening is often performed before levonorgestrel intrauterine system insertion.^32^ Recent (>1 year) levonorgestrel intrauterine system use but not etonogestrel implant or depot-medroxyprogesterone acetate use was associated with decreased cervical cancer risk. Recent levonorgestrel intrauterine system and etonogestrel implant use but not depot-medroxyprogesterone acetate use was also associated with increased risk of thyroid cancer and melanoma. Both cancers are subject to overdiagnosis,^33^ so observed associations could reflect increased testing among women who choose these contraceptives. Some of this variation may be caused by confounding by factors that increase cancer risk and the type of long-acting, reversible contraceptive used. Except for breast cancer, there was no increased risk of cancer among former levonorgestrel intrauterine system or etonogestrel implant users, and substantial reductions in risk were seen for endometrial cancer. Consequently, there was no association between former etonogestrel implant users (>2 years since last use) and cancer risk overall; however, we did not see the same risk reduction as we did for former oral contraceptive pill users. Former levonorgestrel intrauterine system users still had an 11% increased risk of developing cancer, but longer follow-up is needed to determine whether this association will weaken further with longer periods since stopping use.

In contrast, depot-medroxyprogesterone acetate was not associated with breast cancer but was associated with a reduced risk of endometrial cancer and increased risks of cervical, colorectal, lung, and the combined group of “other” cancers, although it is likely that the apparent associations with lung cancer (and perhaps other cancers) were at least partially due to uncontrolled confounding by smoking. One explanation for the breast cancer association is that depot-medroxyprogesterone acetate suppresses ovarian function and circulating estrogen to a greater extent than the levonorgestrel intrauterine system and etonogestrel implant,^34-36^ which may mean that synergistic effects of estrogen and progestin on breast epithelial cell proliferation may be reduced during depot-medroxyprogesterone acetate use.^37^

Our findings are consistent with prior studies showing associations between long-acting, reversible contraceptive use and increased breast cancer^13^^,^^38^ and decreased ovarian cancer risk^14-16^; however, we also investigated other cancer types as well as duration and timing of use. We compared the associations with long-acting, reversible contraceptives with those for the oral contraceptive pill; these associations were consistent with prior studies showing a short-term increase for breast cancer but long-term reductions in risk for endometrial and epithelial ovarian cancers.^8^^,^^10^ With the exception of melanoma, there was no increased risk of other specific cancers among former users and, as a result, former oral contraceptive pill use was associated with a 7% reduced risk of all cancers combined. A previous study of oral contraceptive pill use and melanoma noted that sunburn and use of sunbeds were higher among oral contraceptive pill users,^39^ suggesting that this association could be the result of residual confounding.

To our knowledge, this is the first study to investigate the timing and duration of levonorgestrel intrauterine system, etonogestrel implant, and depot-medroxyprogesterone acetate use as well as associations with overall cancer risk and a wide range of individual cancer types. We had a large sample size and accurate medication dispensing histories. Our findings highlighted that timing and duration of use are important when considering associations between long-acting, reversible contraceptive use and cancer risk.

The major limitations of our study are the lack of data for depot-medroxyprogesterone acetate and oral contraceptive pill use before July 2002 and the limited information about potential confounders. In the early years of follow-up, this limitation will have led to some long-term depot-medroxyprogesterone acetate and oral contraceptive pill users being misclassified as short-term users and former users as never users. Our sensitivity analyses among subgroups where misclassification would be less likely did not materially alter our results, suggesting that they were not seriously biased. We could not determine whether the levonorgestrel intrauterine system or etonogestrel implant were removed early, so we may have overestimated durations of use, hence underestimating the strength of associations. Confounding by indication for each long-acting, reversible contraceptive is possible, and a woman’s reason for using a specific long-acting, reversible contraceptive may be related to her cancer risk. Our sensitivity analyses among subgroups for whom we had information about smoking, parity, and obesity, however, showed that adjusting for these factors in our main analysis would have been unlikely to materially change our findings. Although underreporting of smoking status from hospital records may have affected our ability to adjust for smoking status, our quantitative bias analysis suggests that this was not a major issue. Multiple comparisons were made in our analyses; therefore, it is possible that some associations arose by chance. We were unable to investigate associations for subtypes of hormonally driven cancer due to data limitations (breast cancer) or small numbers (endometrial and ovarian cancers). Finally, the relatively recent introduction of the levonorgestrel intrauterine system and etonogestrel implant means that we did not have information for women older than 70 years of age at cancer diagnosis and so could not explore the potential for further long-term reductions in gynecological cancer risk. Follow-up beyond 10 years from initial use is required before conclusions can be drawn about long-term harms and benefits.

Our study suggests that, like the oral contraceptive pill, long-acting, reversible contraceptives—particularly the levonorgestrel intrauterine system and etonogestrel implant—are associated with reduced endometrial and ovarian cancer risk and increased breast cancer risk. This finding suggests that temporal changes in contraceptive use are unlikely to have a substantial impact on rates of cancer affected by contraceptive use. This information may be helpful to women and their physicians when discussing contraception options.

## Supplementary Material

djae282_Supplementary_Data

## Data Availability

The data underlying this article cannot be shared for the privacy of individuals who participated in the study. Summary-level data can be shared on request to the corresponding author, K.M.T. Individual-level data can be accessed only from within Australia by named project staff with data custodian and Human Research Ethics Committee approval. Please contact K.M.T. for further information.
