# Supplementary material for: Long-acting, progestin-based contraceptives and risk of breast, gynecological, and other cancers
Source: J Natl Cancer Inst. 2025 Jan 14;117(5):1046–55. doi: 10.1093/jnci/djae282 (PMC12058256; doi:10.1093/jnci/djae282)
Supplement: djae282_Supplementary_Data [file djae282_supplementary_data.pdf]

**Supplementary Materials**

This document contains supplementary materials for the following manuscript "*Long-acting, progestin-based contraceptives and risk of breast, gynaecological and other cancers.*"

**Table of Contents**


List of Supplementary Tables ........................................ 1

Supplementary methods ........................................ 3

Area-level socioeconomic status and remoteness categories ........................................ 3

Concessional sub-cohort ........................................ 3

Rx-Risk Comorbidity Score ........................................ 4

Obesity prediction model ........................................ 4

Sensitivity Analyses for Western Australian women ........................................ 5

References ........................................ 6

Supplementary Tables ........................................ 7

Supplementary Figure ........................................ 18


**List of Supplementary Tables**


Supplementary Table 1: Topography codes for cancer subgroups ........................................ 7

Supplementary Table 2: Cancers included in the 'other' cancers group ........................................ 7

Supplementary Table 3: ATC codes for the medicines of interest ........................................ 8

Supplementary Table 4: Hospital procedure and diagnosis codes for Western Australian women ........................................ 9

Supplementary Table 5: Subset of Rx-Risk Comorbidity categories, weights and ATC Codes included in the Rx-Risk comorbidity index. ........................................ 10

Supplementary Table 6: Characteristics of cases in the main analysis by cancer type ........................................ 11

Supplementary Table 7: Characteristics of cases in the concessional sub-cohort by cancer type ........................................ 12

Supplementary Table 8: Associations between LARCs and risk of any cancer by age at diagnosis (concessional cohort) ........ 13

Supplementary Table 9: Association between LARCs and breast cancer: concessional overall, for younger women and for longer PBS history. ........ 14

Supplementary Table 10: Sensitivity Analysis: Western Australian women medicine use and risk of cancer ........ 15

Supplementary Table 11: Sensitivity analysis adjusting for predicted obesity ........ 16

Supplementary Table 12: Association between long-acting, progestin-based contraceptives and breast cancer risk with no contraception use as the reference (concessional sub-cohort) ........ 17

**Supplementary methods**

*Area-level socioeconomic status and remoteness categories*

We used postcode at Medicare enrolment to assign each case and control to a state of residence, area-level socioeconomic status and remoteness category. We estimated area-level socioeconomic status quintiles using the Socio-Economic Indexes for Areas (SEIFA) Index of Relative Socio-Economic Disadvantage published by the Australian Bureau of Statistics (ABS)[1]. We classified remoteness of residence using the Accessibility/Remoteness Index of Australia (ARIA), including major cities, inner regional, outer regional, remote/very remote.[2] We used the earliest available SEIFA (2001) and ARIA (2006) scores, however if this score was missing, the 2006 or 2011 score was used instead. We were unable to assign SEIFA/ARIA scores to 1,569 cases (<1%, and their matched controls).

*Concessional sub-cohort*

The Pharmaceutical Benefits Scheme (PBS) provides timely, reliable and affordable access to necessary medicines for Australians.[3] Under the PBS, the government subsidises the cost of medicine for most medical conditions. All Australian residents registered for Medicare are entitled to a subsidy under the PBS, however Australians eligible for a concession also receive a subsidy for low-cost medicines. Australians who hold a Pensioner concession card, Commonwealth Seniors Health Card, Health Care Card, or Department of Veterans' Affairs card are eligible for a concession. Co-payment (out-of-pocket cost) for concessional beneficiaries for each PBS medicine ranged from $3.60 to $5.90 in 2002 to 2013, with the remaining cost of the medicine paid for by the Australian Government. The co-payment for general beneficiaries, however, ranged from $22.40 to $36.10 during this time.[3] Prior to 2012, only dispensed medicines that attracted an Australian government subsidy were recorded on the PBS records. Prior to 2012, the PBS records only include dispensed medicines that attracted an Australian government subsidy, but all dispensed PBS medicines are included after 2012. The cost of many medicines is above the co-payment amount for both general and concessional beneficiaries, and therefore the PBS records include most prescribed medicines from 2002 to 2013. The levonorgestrel intrauterine system (LNG-IUS) and etonogestrel implants (ENG-IMP) were above co-payment throughout our study period and therefore would be captured by PBS records for all women in our study. However, for depot-medroxyprogesterone acetate (DMPA), menopausal hormone therapy (MHT) and the oral contraceptive pill (OCP), most items are below co-payment for general beneficiaries, so these might not be recorded for general beneficiaries prior to 2012. In addition, private OCP prescriptions are not included in the PBS. We therefore identified a sub-cohort of women who were concessional beneficiaries and thus would have most

hormone use captured for the full study period. Women became eligible for inclusion in this sub-cohort after a minimum of two calendar years recorded as a concessional beneficiary in the PBS (their concessional eligibility date). A woman remained eligible until she had only general prescriptions for a calendar year. If a woman had no medicines dispensed in a calendar year, she retained her status from the previous year.

*Rx-Risk Comorbidity Score*

We estimated comorbidity using the validated weighted Rx-Risk Comorbidity Score (Rx-Risk score),[4] which has been mapped to Australian PBS item codes.[5] Our dataset included all PBS item numbers that were relevant to our study, plus a number of additional medicines although, as described in the Concessional sub-cohort section above, some low-cost medicines that fall under the government co-payment threshold may be under ascertained for general beneficiaries prior to 2012. We could use the PBS data to ascertain most comorbidities included in the Rx-Risk score calculation, with most of those missing having either low or zero weighting or for rare conditions and unlikely to materially affect the score. Supplementary Table 5 provides the details of the included comorbidities for the calculated Rx-Risk score. We used dispensing records from six-months prior to the index date back to two-years prior to calculate the Rx-Risk score for each woman.

*Obesity prediction model*

We used a model previously developed to predict obesity from PBS data[6] for women who were concessional beneficiaries for the whole duration of the study (2002-2013) (a subset of the concessional sub-cohort). The prediction model gives a probability score between 0 and 100 for having a body mass index (BMI) of 30kg/m$^2$ or higher and the scores show the expected associations with the cancers of interest, namely a strong positive association with endometrial cancer, a weaker association for post-menopausal breast cancer and inverse association for pre-menopausal breast cancer and cervical cancer (Supplementary Table 11). We ran the model for each woman in 2005, 2008 and 2011, using only PBS dispensing histories before and up to that year. We used the obesity prediction score for each case and control for the year of or closest prior to the index date. To assess the likely confounding effects of obesity on our primary estimates, we first ran models identical to our main analysis for breast, endometrial, EOC and cervical cancer among the subgroup of women with predicted obesity values. We then repeated these models including the obesity prediction score as a continuous variable. The results are shown in Supplementary Table 11, which also includes the

number of cases and controls included for each cancer type and the proportions of women with predicted obesity (a score of 50 or higher).

*Sensitivity Analyses for Western Australian women*

Western Australia (WA), the 4th most populous Australian state, has comprehensive historic health data sets that for some variables reach back as far as 1950. The WA Data Linkage Unit provided additional data for this study from the electoral roll, Hospital Morbidity Data System (HDMS) and Emergency Department Data Collection (EDDC), Midwives Notifications (1980-2016) and Birth Registrations (1950-1979).

We used electoral roll information and Medicare registrations to identify women who were residents of WA. The HDMS and EDDC provided data on procedures and diagnoses, including hysterectomy, oophorectomy, and smoking (ICD codes included in Supplementary Table 4) and we estimated parity (0, 1, 2, 3, or 4+) using Births and Midwives data. We selected matched WA controls for the WA cancer cases using the same methods as our main analysis (WA subset 1), and then selected a second set of controls excluding women who had a BSO prior to the index date and including parity in the matching criteria (WA Subset 2). We performed a sensitivity analysis for breast, endometrial, epithelial ovarian and cervical cancer additionally adjusting for parity and smoking using Subset 1, and for smoking, hysterectomy and unilateral-salpingo oophorectomy using Subset 2. For endometrial cancer we additionally also removed women with a hysterectomy from selection in Subset 2. For lung, other and all cancer we performed a sensitivity analysis using Subset 1, additionally adjusting for smoking (Supplementary Table 10).

**References**


1. Australian Bureau of Statistics. Socio-Economic Indexes for Areas (SEIFA) Australia, 2011 [Internet]. Australian Bureau of Statistics. 2011 [cited 19 September 2019]. Available from: http://www.abs.gov.au/websitedbs/censushome.nsf/home/seifa2011?opendocument&navpos=260.

2. Australian Bureau of Statistics, Geography Publications [Internet]. 2017 [cited 19 September 2019]. Available from: http://www.abs.gov.au/websitedbs/D3310114.nsf/home/ABS+Geography+Publications.

3. Australian Government Department of Health and Aged Care, The Pharmaceutical Benefits Scheme, Fees, Patient Contributions and Safety Net Thresholds 2022 [Available from: https://www.pbs.gov.au/info/healthpro/explanatory-notes/front/fee.

4. Lu CY, Barratt J, Vitry A, Roughead E. Charlson and Rx-Risk comorbidity indices were predictive of mortality in the Australian health care setting. J Clin Epidemiol. 2011;64(2):223-8

5. Pratt NL, Kerr M, Barratt JD, Kemp-Casey A, Kalisch Ellett LM, Ramsay E, Roughead EE. The validity of the Rx-Risk Comorbidity Index using medicines mapped to the Anatomical Therapeutic Chemical (ATC) Classification System. BMJ Open. 2018;8(4):e021122

6. Ali S, Na R, Waterhouse M, Jordan SJ, Olsen CM, Whiteman DC, Neale RE. Predicting obesity and smoking using medication data: A machine-learning approach. Pharmacoepidemiol Drug Saf. 2021

7. Feng JL, Dixon-Suen SC, Jordan SJ, Webb PM. Is there sufficient evidence to recommend women diagnosed with endometrial cancer take a statin: Results from an Australian record-linkage study. Gynecol Oncol. 2021;161(3):858-63

8. Matz M, Coleman MP, Carreira H, Salmeron D, Chirlaque MD, Allemani C, Group CW. Worldwide comparison of ovarian cancer survival: Histological group and stage at diagnosis (CONCORD-2). Gynecol Oncol. 2017;144(2):396-404

**Supplementary Tables**

Supplementary Table 1: Topography codes for cancer subgroups

| Cancer Type | Topography |
|---|---|
| Breast | C50 |
| Endometrial | C54-C55[a] |
| Cervical | C53 |
| Epithelial ovarian | C56.9, C48.0-C48.2, C57.0-C57.4, C57.7-C57.9[b] |
| Colorectal | C18-C20 |
| Lung | C33-C34 |
| Skin (excluding basal cell and squamous cell carcinoma) | C44 |
| Thyroid | C73 |

[a] Includes only endometrial cancers as defined by ICD-O-3 morphology codes.[7]

[b] Includes only epithelial ovarian cancers as defined by ICD-O-3 morphology codes.[8]

Supplementary Table 2: Cancers included in the 'other' cancers group

| Cancer Type | % of other cancers (n=36,523) |
|---|---|
| Bone marrow | 17 |
| Lymph nodes | 15 |
| Lip, oral cavity, pharynx | 9 |
| Kidney | 9 |
| Brain | 7 |
| Pancreas | 6 |
| Unknown primary site | 5 |
| Stomach | 4 |
| Connective, subcutaneous and other soft tissue | 3 |
| Vulva | 3 |
| Liver | 2 |
| Anus | 2 |
| Small intestine | 2 |
| Bladder | 2 |
| Uterine cancer (not endometrial) | 2 |
| Other (all other cancers 1% or less each) | 13 |

Supplementary Table 3: ATC codes for the medicines of interest

| Medicine name | ATC Code |
|---|---|
| **LNG-IUS** | |
| Levonorgestrel | G02BA03 |
| **ENG-IMP** | |
| Etonogestrel | G03AC08 |
| **DMPA** | |
| Medroxyprogesterone | G03AC06 |
| **OCP** | |
| Norethisterone + Ethinylestradiol | G03AA05, G03AB04 |
| Norethisterone + Mestranol | G03AA05 |
| Levonorgestrel + Ethinylestradiol | G03AA07, G03AB03 |
| Norethisterone | G03AC01 |
| Levonorgestrel | G03AC03 |
| **Menopausal Hormone Therapy**[a] | |
| Estradiol | G03CA03 |
| Estriol | G03CA04 |
| Oestrone Sulfate Sodium | G03CA07 |
| Conjugated Estrogens | G03CA57 |
| Estradiol + Norethisterone Acetate | G03FA01 |
| Conjugated Estrogens + Medroxyprogesterone | G03FA12 |
| Oestradiol Valerate and Oestradiol Valerate with Cyproterone Acetate | G03FB |
| Norethisterone Acetate + Estradiol (&) Estradiol | G03FB05 |
| Conjugated Estrogens + Medroxyprogesterone | G03FB06 |
| Oestrogens - Conjugated and Oestrogens - Conjugated with Medroxyprogesterone Acetate | G03FB06 |
| Estradiol (&) Estradiol + Dydrogesterone | G03FB08 |

ATC: Anatomical Therapeutic Chemical, DMPA: depot-medroxyprogesterone acetate, ENG-IMP: etonogestrel 68 mg implants, LNG-IUS: levonorgestrel intrauterine system, OCP: combined oral contraceptive

[a] Excludes creams and pessaries.

Supplementary Table 4: Hospital procedure and diagnosis codes for Western Australian women

| Procedure | ICD-10 | ICD-9 | ICPM | COSA |
|---|---|---|---|---|
| Bilateral salpingo-oophorectomy | 3563803, 3571701, 3563812, 3571704, 3571705, 3565303, 3567301, 3575301, 3575602 | 655, 656, 688 | 5654, 5655, 5697 | 0671, 0068, B681, 0690 |
| Unilateral salpingo-oophorectomy | 3563811, 3571311, 3565302, 3567300, 3575300, 3575601, 3563800, 3563801, 3563802, 3571305, 3571305, 3571306, 3571307 | 653, 654, 6522, 6529 | 5651-5653 | A681, 0672, A672 |
| Salpingo-oophorectomy non-specified [a] | 3565304, 3566100, 3566400, 3566401, 3566700, 3566701, 3567000, 3567302, 3575302, 3575603, 9044802 | | 5665 | 0673, 0691, 0681 |
| Hysterectomy | 3565300-3565304, 3565700, 3565800, 3566100, 3566400, 3566401, 3566700, 3566701, 3567000, 3567300-3567302, 3575000, 3575300-3575302, 3575600-3575603, 9044300, 9044800-9044802 | 683-689 | 5682-5687 | 0690, A690, B690, C690, 0691, 0692, A692, 0693, A693, B693, C693, 0694, 0696, 0744, 0764 |
| **Diagnosis** | **ICD-10** | **ICD-9** | **ICPM** | **COSA** |
| Smoking[b] | | | | |
| Use of tobacco past or current | Z720 | V1582 | | |
| Mental/behavioral disorders due to smoking | F17, Z8643 | 3051 | | |
| Treatment for tobacco disorders | Z508, Z716 | | | |

COSA: Code of Surgical Operations, Commonwealth Department of Health, Canberra 1968; ICD: International Classification of Diseases; ICPM: International Classification of Procedures in Medicine, WHO Geneva 1978.

[a] Classified as unilateral salpingo-oophorectomy where a woman also received another salpingo-oophorectomy procedure, otherwise classified as a bilateral salpingo-oophorectomy in our analyses.

[b] We used diagnosis codes from hospital records to categorize smoking status as never smoked (no smoking diagnosis codes), ever smoked (any smoking diagnosis codes).

Supplementary Table 5: Subset of Rx-Risk Comorbidity categories, weights and ATC Codes included in the Rx-Risk comorbidity index.

| Rx-Risk Comorbidity Category[a] | Weight for Rx-Risk score | ATC Codes | Cases | | Controls | |
|---|---|---|---|---|---|---|
| | | | n | % | n | % |
| Anticoagulants | 1 | B01AA03-B01AB06, B01AE07, B01AF01, B01AF02, B01AX05 | 5,264 | 3 | 22,577 | 3 |
| Antiplatelets | 2 | B01AC04–B01AC30 | 5,331 | 3 | 23,070 | 3 |
| Arrhythmia | 2 | C01AA05, C01BA01–C01BD01, C07AA07 | 1,087 | 1 | 4,375 | 0 |
| Congestive heart failure | 2 | C03DA02–C03DA99, C07AB02 (if PBS item code is 8732N, 8733P, 8734Q, 8735R, 08818D), C07AB07, C07AG02, C07AB12, and both of (C03CA01–C03CC01) and (C09AA01–C09AX99, C09CA01–C09CX99) | 2,231 | 1 | 8,248 | 1 |
| Diabetes | 2 | A10AA01–A10BX99 | 7,928 | 4 | 32,487 | 4 |
| Hyperlipidemia | -1 | A10BH03, C10AA01–C10BX09 | 26,028 | 15 | 123,538 | 14 |
| Hypertension | -1 | C03AA01–C03BA11, C03DB01-C03DB99, C03EA01, C09BA02–C09BA09, C09DA02–C09DA08, C02AB01–C02AC05, C02DB02–C02DB99, (C03CA01–C03CCO1) or (C09CA01–C09CX99) but not both groups. | 28,447 | 16 | 126,028 | 14 |
| Hyperthyroidism | 2 | H03BA02, H03BB01 | 1,198 | 1 | 4,593 | 1 |
| Ischemic heart disease: angina | 2 | C01DA02–C01DA14, C01DX16, C08EX02 | 2,150 | 1 | 9,265 | 1 |
| Ischemic heart disease: hypertension | -1 | C07AA01–C07AA06, C07AA08–C07AB03, C07AB02 (if PBS item code is not 8732N, 8733P, 8734Q, 8735R, 08818D) C07AG01, C08CA01–C08DB01, C09DB01–C09DB04, C09DX01, C09BB02–C09BB10, C09DX03, C10BX03 | 14,789 | 8 | 64,415 | 7 |
| Inflammation/pain | -1 | M01AB01–M01AH06 | 35,936 | 20 | 172,073 | 19 |
| Malignancies | 2 | L01AA01–L01XX41 | 2,208 | 1 | 8,766 | 1 |
| Osteoporosis/Paget's | -1 | M05BA01–M05BB05, M05BX03, M05BX04, G03XC01, H05AA02 | 3,226 | 2 | 15,439 | 2 |
| Pain | 3 | N02AA01–N02AX02, N02AX06, N02AX52, N02BE51 | 26,914 | 15 | 122,044 | 14 |
| Pulmonary hypertension | 6 | C02KX01–C02KX05, C02KX | 29 | 0 | 60 | 0 |
| Steroid-responsive disease | 2 | H02AB01–H02AB10 | 12,942 | 7 | 57,771 | 7 |

ATC: Anatomical Therapeutic Chemical

[a] Comorbidity categories excluded from our calculation due to PBS item codes not being available in our data set: Alcohol dependency, Allergies, Anxiety, Bipolar disorder, Dementia, Gout, Hyperkalemia, Liver failure, Migraine, Parkinson's disease, Psychotic Illness, Renal disease, Smoking cessation. For each of these, less than 12% of the Department of Veteran Affairs cohort were identified as having the condition and unlikely to affect the weighted Rx-Risk score.

Supplementary Table 6: Characteristics of cases in the main analysis by cancer type

| **Cancer Type** | All Cancers | Breast | Endometrial | Ovarian | Cervical | Colorectal | Lung | Melanoma | Thyroid | Other |
|---|---|---|---|---|---|---|---|---|---|---|
| Total | 176,601 | 67,470 | 7,290 | 4,752 | 4,755 | 14,558 | 8,504 | 23,171 | 9,578 | 36,523 |
| **Characteristic** [a] | % | % | % | % | % | % | % | % | % | % |
| **Age at diagnosis (years)** | | | | | | | | | | |
| median (range) | 52 (20,66) | 52 (20,66) | 56 (20,66) | 53 (20,66) | 42 (20,66) | 54 (20,66) | 56 (20,66) | 49 (20,66) | 46 (20,66) | 53 (20,66) |
| <30 | 3.8 | 0.8 | 0.4 | 2.4 | 12.4 | 3.0 | 0.7 | 8.1 | 9.1 | 5.9 |
| 30-39 | 10.8 | 8.3 | 4.4 | 6.9 | 30.7 | 7.1 | 2.9 | 17.8 | 21.8 | 10.4 |
| 40-49 | 27.4 | 32.3 | 14.8 | 25.0 | 30.3 | 21.4 | 17.3 | 29.0 | 31.9 | 23.5 |
| 50-59 | 42.8 | 44.5 | 55.7 | 48.4 | 21.9 | 48.2 | 53.1 | 34.5 | 30.0 | 43.4 |
| 60-69 | 15.2 | 14.0 | 24.7 | 17.3 | 4.6 | 20.3 | 26.1 | 10.6 | 7.2 | 16.9 |
| **State** | | | | | | | | | | |
| NSW | 32.9 | 32.7 | 32.5 | 33.7 | 31.2 | 32.1 | 34.2 | 31.8 | 39.6 | 32.4 |
| ACT | 1.6 | 1.8 | 1.6 | 1.9 | 1.2 | 1.6 | 1.4 | 1.5 | 1.4 | 1.5 |
| VIC | 24.2 | 25.0 | 26.3 | 25.5 | 21.6 | 25.4 | 23.7 | 20.8 | 19.9 | 25.3 |
| QLD | 20.7 | 19.4 | 19.9 | 18.1 | 23.3 | 19.4 | 20.5 | 27.1 | 21.8 | 19.5 |
| SA & NT | 8.4 | 8.6 | 9.5 | 9.3 | 9.1 | 8.9 | 8.3 | 6.9 | 5.9 | 9.0 |
| WA | 9.7 | 10.0 | 8.1 | 9.2 | 10.7 | 9.6 | 9.2 | 9.1 | 9.8 | 9.7 |
| TAS | 2.5 | 2.5 | 2.1 | 2.4 | 2.9 | 2.9 | 2.8 | 2.7 | 1.6 | 2.6 |
| **SEIFA Quintile** | | | | | | | | | | |
| 1 [b] | 19.2 | 17.3 | 22.3 | 18.9 | 24.1 | 19.9 | 25.0 | 16.6 | 21.1 | 21.2 |
| 2 | 19.8 | 18.9 | 20.1 | 19.2 | 21.1 | 20.7 | 21.8 | 21.2 | 18.6 | 20.0 |
| 3 | 20.0 | 19.6 | 19.5 | 19.3 | 20.0 | 20.3 | 19.7 | 21.0 | 19.9 | 20.1 |
| 4 | 20.1 | 20.9 | 19.7 | 20.8 | 18.7 | 20.2 | 17.7 | 20.9 | 18.9 | 19.3 |
| 5 | 20.0 | 22.4 | 17.4 | 20.9 | 15.3 | 18.1 | 14.9 | 19.6 | 20.7 | 18.6 |
| missing | 0.9 | 0.9 | 0.9 | 0.9 | 0.8 | 0.8 | 0.9 | 0.8 | 0.8 | 0.9 |
| **Remoteness** | | | | | | | | | | |
| major city | 68.6 | 70.1 | 69.5 | 70.7 | 67.0 | 67.1 | 64.7 | 64.2 | 75.0 | 68.1 |
| inner regional | 19.9 | 19.2 | 19.0 | 18.5 | 19.3 | 20.5 | 21.8 | 23.2 | 15.6 | 20.0 |
| outer regional | 8.9 | 8.3 | 8.8 | 8.4 | 10.3 | 9.8 | 10.1 | 10.2 | 7.1 | 9.1 |
| remote/very remote | 1.8 | 1.6 | 1.8 | 1.5 | 2.6 | 1.8 | 2.5 | 1.8 | 1.6 | 2.0 |
| missing | 0.8 | 0.9 | 0.9 | 0.9 | 0.8 | 0.7 | 0.8 | 0.7 | 0.7 | 0.8 |

[a] Proportions for controls are the same as cases.
[b] Most disadvantaged.

Supplementary Table 7: Characteristics of cases in the concessional sub-cohort by cancer type

| **Cancer Type** | All Cancers | Breast | Endometrial | Ovarian | Cervical | Colorectal | Lung | Melanoma | Thyroid | Other |
|---|---|---|---|---|---|---|---|---|---|---|
| Total | 47,645 | 15,391 | 2,206 | 1,273 | 1,702 | 4,124 | 3,525 | 5,346 | 2,454 | 11,624 |
| **Characteristic** [a] | % | % | % | % | % | % | % | % | % | % |
| **Age at diagnosis (years)** | | | | | | | | | | |
| median (range) | 52 (20,66) | 53 (20,66) | 58 (20,66) | 55 (20,66) | 41 (20,66) | 56 (20,66) | 57 (21,66) | 48 (20,66) | 47 (20,66) | 54 (20,66) |
| <30 | 4.4 | 0.8 | 0.5 | 2.5 | 13.7 | 2.9 | 0.4 | 11.1 | 10.1 | 6.0 |
| 30-39 | 9.9 | 7.4 | 4.2 | 6.7 | 31.0 | 6.0 | 2.6 | 17.8 | 19.2 | 9.3 |
| 40-49 | 24.2 | 28.5 | 11.8 | 22.7 | 28.7 | 18.3 | 16.6 | 27.0 | 31.3 | 21.9 |
| 50-59 | 39.5 | 41.2 | 50.9 | 45.0 | 20.9 | 43.7 | 47.9 | 28.9 | 28.5 | 40.2 |
| 60-69 | 22.1 | 22.1 | 32.7 | 23.1 | 5.6 | 29.1 | 32.4 | 15.1 | 10.8 | 22.6 |
| **State** | | | | | | | | | | |
| NSW | 33.2 | 32.5 | 31.4 | 34.6 | 32.7 | 33.0 | 35.7 | 31.1 | 40.5 | 33.1 |
| ACT | 0.8 | 0.8 | 0.7 | 0.7 | 0.5 | 0.9 | 0.8 | 0.9 | 0.5 | 0.9 |
| VIC | 24.1 | 25.4 | 27.1 | 25.2 | 20.1 | 25.0 | 23.9 | 19.0 | 21.4 | 24.7 |
| QLD | 21.3 | 20.2 | 20.9 | 18.7 | 23.9 | 19.8 | 20.3 | 29.4 | 19.8 | 20.0 |
| SA & NT | 8.6 | 8.8 | 10.1 | 9.3 | 9.2 | 9.4 | 7.9 | 7.4 | 6.0 | 8.9 |
| WA | 8.8 | 9.2 | 7.2 | 7.9 | 9.3 | 7.9 | 7.7 | 8.4 | 9.8 | 9.1 |
| TAS | 3.4 | 3.2 | 2.7 | 3.5 | 4.2 | 3.9 | 3.7 | 4.0 | 1.9 | 3.3 |
| **SEIFA** | | | | | | | | | | |
| 1[b] | 29.4 | 27.5 | 31.7 | 30.6 | 34.2 | 29.1 | 33.7 | 25.6 | 32.9 | 30.2 |
| 2 | 24.0 | 23.6 | 23.3 | 22.3 | 25.0 | 23.9 | 23.6 | 26.7 | 22.1 | 24.0 |
| 3 | 20.3 | 20.4 | 18.4 | 20.9 | 19.7 | 20.8 | 20.5 | 21.5 | 19.4 | 20.0 |
| 4 | 15.9 | 16.8 | 17.7 | 15.9 | 13.7 | 16.1 | 13.8 | 16.3 | 14.0 | 15.6 |
| 5 | 9.8 | 11.0 | 8.4 | 9.6 | 6.8 | 9.6 | 7.8 | 9.3 | 10.8 | 9.6 |
| missing | 0.6 | 0.7 | 0.6 | 0.7 | 0.5 | 0.6 | 0.5 | 0.7 | 0.8 | 0.6 |
| **Remoteness** | | | | | | | | | | |
| major city | 62.8 | 63.8 | 65.6 | 62.5 | 62.9 | 63.3 | 60.3 | 55.7 | 72.2 | 63.0 |
| inner regional | 24.3 | 24.2 | 21.7 | 23.4 | 23.1 | 22.8 | 26.0 | 29.3 | 18.1 | 24.2 |
| outer regional | 10.6 | 10.0 | 10.4 | 12.2 | 11.2 | 11.7 | 11.1 | 12.9 | 7.7 | 10.5 |
| remote/very remote | 1.6 | 1.4 | 1.8 | 1.2 | 2.3 | 1.6 | 2.0 | 1.5 | 1.3 | 1.8 |
| missing | 0.6 | 0.6 | 0.5 | 0.7 | 0.5 | 0.6 | 0.5 | 0.6 | 0.7 | 0.6 |

[a] Proportions for controls are the same as cases.
[b] Most disadvantaged.

Supplementary Table 8: Associations between LARCs and risk of any cancer by age at diagnosis (concessional cohort)

| | **Age at diagnosis: <40 years** | | | **40-49 years** | | | **50-59 years** | | | **60-67 years** | | |
|---|---|---|---|---|---|---|---|---|---|---|---|---|
| | Cases **%** | Controls **%** | OR (95% CI) | Cases % | Controls % | OR (95% CI) | Cases **%** | Controls **%** | OR (95% CI) | Cases % | Controls % | OR (95% CI) |
| **LNG-IUS** | | | | | | | | | | | | |
| No use | 93.3 | 94.5 | Reference | 93.4 | 94.7 | Reference | 97.6 | 98.2 | Reference | 99.4 | 99.7 | Reference |
| Recent user (1+years) | 6.5 | 5.3 | 1.20 (1.06,1.36) | 6.3 | 5.0 | 1.15 (1.04,1.27) | 2.1 | 1.5 | 1.22 (1.08,1.39) | 0.5 | 0.2 | 1.49 (1.01,2.20) |
| Former user | 0.2 | 0.3 | 0.70 (0.38,1.28) | 0.4 | 0.3 | 1.10 (0.79,1.55) | 0.3 | 0.3 | 1.14 (0.86,1.51) | 0.1 | 0.1 | 1.36 (0.75,2.47) |
| **ENG-IMP** | | | | | | | | | | | | |
| No use | 88.4 | 89.4 | Reference | 95.7 | 96.1 | Reference | 99.4 | 99.5 | Reference | 99.9 | 99.9 | Reference |
| Recent user (1+years) | 8.3 | 7.3 | 1.13 (1.02,1.25) | 2.8 | 2.4 | 1.14 (1.00,1.30) | <0.5 | 0.2 | 1.01 (0.73,1.39) | <0.1 | <0.1 | 0.97 (0.11,8.33) |
| Former user | 3.3 | 3.3 | 0.99 (0.85,1.15) | 1.5 | 1.4 | 1.02 (0.87,1.21) | <0.5 | 0.3 | 1.04 (0.79,1.37) | <0.1 | <0.1 | 1.30 (0.43,3.93) |
| **DMPA** | | | | | | | | | | | | |
| No use | 88.2 | 89.6 | Reference | 93.7 | 94.5 | Reference | 98.5 | 98.6 | Reference | 99.9 | 99.8 | Reference |
| Recent user (1+years) | 6.3 | 5.3 | 1.30 (1.14,1.49) | 3.2 | 2.7 | 1.14 (1.00,1.31) | <0.5 | 0.4 | 1.07 (0.81,1.41) | - | - | - |
| Former user | 5.5 | 5.2 | 1.08 (0.88,1.32) | 3.1 | 2.8 | 1.24 (1.03,1.50) | <1.5 | 1.0 | 1.11 (0.89,1.40) | <0.2 | 0.2 | 0.44 (0.16,1.21) |
| **OCP** | | | | | | | | | | | | |
| No use | 61.7 | 63.1 | Reference | 78.9 | 79.8 | Reference | 93.9 | 94.0 | Reference | 98.9 | 99.0 | Reference |
| Recent user (1+years) | 23.1 | 20.1 | 1.16 (1.08,1.24) | 12.0 | 10.2 | 1.23 (1.15,1.31) | 2.2 | 1.7 | 1.40 (1.25,1.57) | 0.2 | 0.1 | 1.48 (0.80,2.76) |
| Former user | 15.3 | 16.8 | 0.96 (0.87,1.06) | 9.2 | 10.0 | 0.92 (0.83,1.01) | 3.9 | 4.3 | 0.90 (0.81,1.00) | 0.9 | 0.9 | 0.95 (0.70,1.28) |
| **Total** | **6,772** | **33,848** | | **11,521** | **57,585** | | **18,800** | **93,928** | | **10,551** | **52,746** | |

CI: Confidence Interval, DMPA: depot-medroxyprogesterone acetate, ENG-IMP: etonogestrel 68 mg implants, LNG-IUS: levonorgestrel intrauterine system, OCP: combined oral contraceptive, OR: Odds Ratio

All models adjusted for weighted Rx-Risk score at index date and menopausal hormone therapy, and matched by age, SEIFA, remoteness, and registered state; and include all contraceptives in the same model.

Supplementary Table 9: Association between LARCs and breast cancer: concessional overall, for younger women and for longer PBS history.

| | Concessional | | | Women <45 years in 2002 | | | Follow-up commenced 2008 | | |
|---|---|---|---|---|---|---|---|---|---|
| Medicine use [a] | Cases | Controls | OR (95% CI) [b] | Cases | Controls | OR (95% CI) [b] | Cases | Controls | OR (95% CI) [b] |
| **LNG-IUS** | | | | | | | | | |
| No use | 96.2 | 97.2 | Reference | 92.7 | 94.4 | Reference | 95.2 | 96.6 | Reference |
| Any use | 3.8 | 2.8 | 1.37 (1.25,1.51) | 7.3 | 5.2 | 1.31 (1.18,1.46) | 4.8 | 3.4 | 1.45 (1.31,1.60) |
| Use category: | | | | | | | | | |
| Recent: short-term | 0.5 | 0.4 | 1.21 (0.94,1.56) | 1.1 | 0.8 | 1.27 (0.97,1.65) | 0.6 | 0.4 | 1.36 (1.02,1.81) |
| Recent: medium-term | 2.1 | 1.5 | 1.42 (1.25,1.61) | 4.0 | 3.1 | 1.37 (1.19,1.57) | 2.5 | 1.7 | 1.52 (1.32,1.74) |
| Recent: long-term | 0.9 | 0.6 | 1.49 (1.22,1.81) | 1.7 | 1.3 | 1.41 (1.13,1.74) | 1.2 | 0.9 | 1.50 (1.23,1.82) |
| Former user | 0.4 | 0.3 | 1.47 (1.09,1.97) | 0.6 | 0.5 | 1.28 (0.89,1.85) | 0.5 | 0.4 | 1.47 (1.09,1.97) |
| **ENG-IMP** | | | | | | | | | |
| No use | 97.5 | 97.9 | Reference | 94.5 | 95.3 | Reference | 97.1 | 97.7 | Reference |
| Any use | 2.5 | 2.1 | 1.16 (1.03,1.30) | 5.5 | 4.7 | 1.15 (1.02,1.30) | 2.9 | 2.3 | 1.20 (1.06,1.37) |
| Use category: | | | | | | | | | |
| Recent: short-term | 0.2 | 0.2 | 1.07 (0.71,1.60) | 0.4 | 0.4 | 1.07 (0.71,1.61) | 0.1 | 0.1 | 1.05 (0.61,1.82) |
| Recent: medium-term | 1.0 | 0.9 | 1.10 (0.92,1.32) | 2.1 | 1.9 | 1.05 (0.87,1.27) | 0.9 | 0.7 | 1.19 (0.95,1.50) |
| Recent: long-term | 0.5 | 0.3 | 1.69 (1.29,2.23) | 1.1 | 0.7 | 1.71 (1.30,2.26) | 0.6 | 0.4 | 1.68 (1.28,2.22) |
| Former user | 0.9 | 0.8 | 1.11 (0.92,1.34) | 2.0 | 1.8 | 1.12 (0.92,1.36) | 1.2 | 1.1 | 1.09 (0.90,1.31) |
| **DMPA** | | | | | | | | | |
| No use | 97 | 97 | Reference | 93.7 | 93.9 | Reference | 96.6 | 96.9 | Reference |
| Any use | 3.1 | 3 | 1.01 (0.91,1.12) | 6.3 | 6.1 | 1.01 (0.91,1.13) | 3.4 | 3.1 | 1.07 (0.95,1.21) |
| Use category: | | | | | | | | | |
| Recent: short-term | 0.3 | 0.4 | 0.89 (0.66,1.19) | 0.7 | 0.7 | 0.89 (0.65,1.23) | 0.3 | 0.3 | 1.04 (0.70,1.54) |
| Recent: medium-term | 0.5 | 0.5 | 1.00 (0.78,1.28) | 1.1 | 1.0 | 1.04 (0.80,1.35) | 0.3 | 0.3 | 1.18 (0.81,1.73) |
| Recent: long-term | 0.5 | 0.4 | 1.23 (0.95,1.59) | 1.1 | 0.9 | 1.24 (0.95,1.62) | 0.6 | 0.6 | 1.24 (0.96,1.61) |
| Former: short-term | 1.1 | 1.1 | 0.98 (0.83,1.16) | 2.1 | 2.2 | 0.95 (0.79,1.14) | 1.3 | 1.2 | 1.00 (0.83,1.20) |
| Former user (1+ years) | 0.7 | 0.6 | 1.08 (0.87,1.34) | 1.4 | 1.2 | 1.10 (0.87,1.38) | 0.9 | 0.8 | 1.11 (0.89,1.39) |
| **OCP** | | | | | | | | | |
| No use | 86.5 | 88.5 | Reference | 74.1 | 77.6 | Reference | 86.2 | 88.4 | Reference |
| Any use | 13.5 | 11.5 | 1.24 (1.17,1.31) | 25.9 | 22.4 | 1.22 (1.15,1.30) | 13.8 | 11.6 | 1.25 (1.18,1.34) |
| Use category: | | | | | | | | | |
| Recent: short-term | 1.0 | 0.8 | 1.26 (1.05,1.51) | 2.1 | 1.7 | 1.34 (1.10,1.62) | 0.7 | 0.5 | 1.59 (1.23,2.07) |
| Recent: medium-term | 3.9 | 2.7 | 1.51 (1.37,1.67) | 7.6 | 5.3 | 1.52 (1.36,1.69) | 2.2 | 1.3 | 1.78 (1.53,2.07) |
| Recent: long-term | 2.7 | 1.7 | 1.70 (1.51,1.92) | 5.6 | 3.7 | 1.60 (1.41,1.82) | 3.7 | 2.4 | 1.71 (1.52,1.93) |
| Former: short-term | 2.6 | 2.8 | 0.98 (0.88,1.10) | 4.7 | 5.2 | 0.94 (0.83,1.07) | 2.9 | 3.1 | 1.00 (0.88,1.13) |
| Former user (1+ years) | 3.4 | 3.5 | 1.04 (0.94,1.14) | 6.0 | 6.4 | 0.98 (0.88,1.10) | 4.3 | 4.4 | 1.04 (0.94,1.16) |
| **Total** | **15,391** | **76,912** | | **6,600** | **33,023** | | **11,087** | **55,392** | |

CI: Confidence Interval, DMPA: depot-medroxyprogesterone acetate, ENG-IMP: etonogestrel 68 mg implants, LNG-IUS: levonorgestrel intrauterine system, OCP: combined oral contraceptive, OR: Odds Ratio, SEIFA: Socio-Economic Indexes for Areas.

[a] Short-term use: <12 months; medium-term use: 1-<5 years; long-term use: 5 or more years.

[b] All models adjusted for weighted Rx-Risk score at index date and menopausal hormone therapy, and matched by age, SEIFA, remoteness, and registered state; and include all contraceptives in the same model.

Supplementary Table 10: Sensitivity Analysis: Western Australian women medicine use and risk of cancer

| | WA Subset 1[a] | | | | WA Subset 2[b] | | | | |
|---|---|---|---|---|---|---|---|---|---|
| | Cases % | Controls % | Analysis 1 OR (95% CI) | Analysis 2[c] OR (95% CI) | Cases % | Controls % | Analysis 3 OR (95% CI) | Analysis 4[d] OR (95% CI) | Analysis 5[e] OR (95% CI) |
| **Breast cancer** | | | | | | | | | |
| LNG-IUS use | 4.3 | 4.0 | 1.06 (0.92,1.22) | 1.05 (0.91,1.21) | 4.4 | 3.9 | 1.14 (0.98,1.32) | 1.13 (0.97,1.30) | 1.12 (0.97,1.30) |
| ENG-IMP use | 2.2 | 1.7 | 1.30 (1.06,1.59) | 1.24 (1.01,1.51) | 2.3 | 1.8 | 1.25 (1.02,1.53) | 1.22 (0.99,1.49) | 1.22 (0.99,1.49) |
| DMPA use | 1.1 | 0.9 | 1.10 (0.83,1.46) | 1.02 (0.77,1.35) | 1.1 | 0.9 | 1.19 (0.90,1.57) | 1.08 (0.81,1.43) | 1.08 (0.81,1.43) |
| OCP use | 5.8 | 4.6 | 1.29 (1.14,1.47) | 1.27 (1.12,1.45) | 6.0 | 4.7 | 1.29 (1.14,1.47) | 1.28 (1.12,1.45) | 1.27 (1.12,1.45) |
| Total (n) | 5,841 | 29,200 | | | 5,679 | 28,379 | | | |
| **Endometrial cancer** | | | | | | | | | |
| LNG-IUS use | 4.7 | 2.8 | 1.89 (1.14,3.12) | 1.92 (1.15,3.20) | 4.8 | 2.5 | 1.99 (1.21,3.26) | 1.94 (1.19,3.18) | 1.88 (1.15,3.09) |
| ENG-IMP use | <2[f] | 0.9 | 0.24 (0.03,1.83) | 0.24 (0.03,1.77) | <2[f] | 0.6 | 0.23 (0.03,1.78) | 0.23 (0.03,1.78) | 0.24 (0.03,1.82) |
| DMPA use | - | 0.9 | [g] | [g] | - | 0.4 | [g] | [g] | [g] |
| OCP use | <2[f] | 2.7 | 0.35 (0.14,0.90) | 0.37 (0.15,0.96) | <2[f] | 2.8 | 0.33 (0.13,0.84) | 0.32 (0.13,0.81) | 0.32 (0.13,0.81) |
| Total (n) | 506 | 2,530 | | | 502 | 2,507 | | | |
| **Cervical cancer** | | | | | | | | | |
| LNG-IUS use | 3.1 | 5.5 | 0.51 (0.28,0.95) | 0.50 (0.27,0.92) | 2.9 | 5.3 | 0.51 (0.27,0.94) | 0.49 (0.26,0.91) | 0.46 (0.24,0.85) |
| ENG-IMP use | 5.7 | 6.2 | 0.82 (0.52,1.31) | 0.78 (0.48,1.25) | 5.7 | 5.8 | 0.93 (0.58,1.51) | 0.88 (0.54,1.42) | 0.84 (0.52,1.37) |
| DMPA use | 5.5 | 2.1 | 2.56 (1.51,4.36) | 2.33 (1.35,4.02) | 5.5 | 2.5 | 2.21 (1.31,3.75) | 2.13 (1.25,3.63) | 2.16 (1.26,3.68) |
| OCP use | 16.0 | 11.4 | 1.50 (1.08,2.07) | 1.48 (1.06,2.06) | 16.0 | 12.2 | 1.38 (1.01,1.90) | 1.39 (1.01,1.92) | 1.36 (0.99,1.88) |
| Total (n) | 420 | 2,100 | | | 418 | 2,090 | | | |
| **Epithelial ovarian cancer** | | | | | | | | | |
| LNG-IUS use | <2[f] | 3.1 | 0.34 (0.12,0.96) | 0.34 (0.12,0.97) | <2[f] | 2.5 | 0.39 (0.14,1.12) | 0.39 (0.14,1.12) | 0.39 (0.14,1.11) |
| ENG-IMP use | <2[f] | 1.6 | 0.91 (0.34,2.48) | 0.80 (0.29,2.19) | <2[f] | 1.2 | 1.36 (0.49,3.75) | 1.32 (0.48,3.66) | 1.32 (0.48,3.65) |
| DMPA use | <2[f] | 1.0 | 0.27 (0.04,2.08) | 0.28 (0.04,2.16) | <2[f] | 1.1 | 0.23 (0.03,1.75) | 0.23 (0.03,1.70) | 0.23 (0.03,1.68) |
| OCP use | 3.0 | 4.4 | 0.69 (0.35,1.97) | 0.68 (0.34,1.36) | 3.0 | 4.5 | 0.60 (0.30,1.20) | 0.60 (0.30,1.21) | 0.61 (0.30,1.21) |
| Total (n) | 367 | 1,835 | | | 365 | 1,825 | | | |
| **Lung cancer** | | | | | | | | | |
| LNG-IUS use | 2.3 | 2.9 | 0.79 (0.45,1.40) | 0.85 (0.47,1.57) | | | | | |
| ENG-IMP use | 1.1 | 0.9 | 1.18 (0.51,2.74) | 0.93 (0.38,2.31) | | | | | |
| DMPA use | 1.1 | 0.8 | 1.41 (0.59,3.36) | 0.92 (0.36,2.33) | | | | | |
| OCP use | 2.1 | 3.1 | 0.60 (0.33,1.09) | 0.56 (0.30,1.06) | | | | | |
| Total (n) | 661 | 3,305 | | | | | | | |
| **Other cancers** | | | | | | | | | |
| LNG-IUS use | 3.4 | 3.1 | 1.08 (0.87,1.35) | 1.08 (0.86,1.34) | | | | | |
| ENG-IMP use | 2.5 | 2.3 | 1.10 (0.84,1.43) | 1.06 (0.82,1.39) | | | | | |
| DMPA use | 1.6 | 1.2 | 1.21 (0.87,1.67) | 1.08 (0.77,1.50) | | | | | |
| OCP use | 6.0 | 5.4 | 1.10 (0.92,1.31) | 1.11 (0.93,1.33) | | | | | |
| Total (n) | 3,057 | 15,285 | | | | | | | |
| **All cancer** | | | | | | | | | |
| LNG-IUS use | 4.1 | 3.8 | 1.05 (0.96,1.15) | 1.04 (0.95,1.14) | | | | | |
| ENG-IMP use | 2.6 | 2.2 | 1.17 (1.04,1.32) | 1.12 (1.00,1.27) | | | | | |
| DMPA use | 1.3 | 1.1 | 1.12 (0.96,1.31) | 1.03 (0.88,1.21) | | | | | |
| OCP use | 6.0 | 5.4 | 1.11 (1.02,1.20) | 1.10 (1.02,1.19) | | | | | |
| Total (n) | 14,782 | 73,896 | | | | | | | |

CI: Confidence Interval, DMPA: depot-medroxyprogesterone acetate, ENG-IMP: etonogestrel 68 mg implants, LNG-IUS: levonorgestrel intrauterine system, OCP: combined oral contraceptive, OR: Odds Ratio, WA: Western Australia.

[a] WA Subset 1: Eligibility for selection as a case or control was as per the main analysis. Models adjusted for weighted Rx-Risk score and menopausal hormone therapy use, and all medicine use types are included in the same model, with no use as the reference.

[b] WA Subset 2: Women with a bilateral-salpingo oophorectomy were not eligible for selection as a case or control after the date of surgery. Additionally matched by parity category (0,1,2,3,4+). For endometrial cancer, women with a hysterectomy were also excluded from selection as a case or control after the date of surgery. Adjusted for weighted Rx-Risk score and menopausal hormone therapy use, and all medicine use types are included in the same model, with no use as the reference.

[c] Additionally adjusted for parity category (0,1,2,3,4+) and smoking (ever vs never).

[d] Additionally adjusted for smoking (ever vs never).

[e] Additionally adjusted for smoking (ever vs never), hysterectomy and unilateral-salpingo oophorectomy.

[f] Due to small numbers, the percentage has been suppressed.

[g] There were no cases who were DMPA users.

Supplementary Table 11: Sensitivity analysis adjusting for predicted obesity

| | Cases % | Controls % | Primary model [a] OR (95% CI) | Adjusted for obesity [b] OR (95% CI) |
|---|---|---|---|---|
| **Associations between predicted obesity score and cancer [c]** | | | | |
| Breast cancer 50+ yrs [d] | 26.6 | 26.1 | 1.21 (1.06,1.38) | |
| Breast cancer <50 yrs [e] | 11.7 | 12.4 | 0.88 (0.67,1.18) | |
| Endometrial cancer | 41.9 | 29.5 | 4.22 (3.18,5.60) | |
| Ovarian cancer | 26.1 | 26.3 | 1.10 (0.74,1.63) | |
| Cervical cancer | 13.7 | 17.0 | 0.63 (0.39,1.00) | |
| **Associations between contraceptives and cancer risk** | | | | |
| **Breast Cancer (n)** | **9,562** | **44,339** | | |
| LNG-IUS any use | 3.0 | 2.2 | 1.42 (1.22,1.65) | 1.42 (1.22,1.64) |
| ENG-IMP any use | 1.8 | 1.8 | 1.06 (0.89,1.27) | 1.06 (0.88,1.26) |
| DMPA any use | 3.1 | 3.1 | 0.89 (0.77,1.03) | 0.89 (0.77,1.03) |
| OCP any use | 11.7 | 9.5 | 1.24 (1.14,1.34) | 1.24 (1.14,1.35) |
| **Endometrial Cancer (n)** | **1,522** | **6,965** | | |
| LNG-IUS any use | 6.4 | 1.3 | 5.50 (3.84,7.88) | 5.26 (3.65,7.57) |
| ENG-IMP any use | <1.0 | 1.0 | 0.27 (0.08,0.86) | 0.29 (0.09,0.91) |
| DMPA any use | 1.1 | 1.8 | 0.58 (0.33,1.03) | 0.54 (0.30,0.96) |
| OCP any use | 3.0 | 5.9 | 0.41 (0.29,0.58) | 0.43 (0.30,0.62) |
| **EOC (n)** | **916** | **3,836** | | |
| LNG-IUS any use | 2.1 | 2.1 | 0.87 (0.50,1.51) | 0.87 (0.50,1.51) |
| ENG-IMP any use | 1.6 | 1.7 | 0.86 (0.45,1.62) | 0.86 (0.45,1.62) |
| DMPA any use | 2.3 | 2.6 | 0.65 (0.38,1.10) | 0.65 (0.38,1.10) |
| OCP any use | 7.0 | 7.7 | 0.75 (0.54,1.02) | 0.75 (0.54,1.02) |
| **Cervical Cancer (n)** | **1,061** | **4,231** | | |
| LNG-IUS any use | 4.2 | 4.3 | 0.93 (0.65,1.34) | 0.95 (0.66,1.37) |
| ENG-IMP any use | 6.9 | 5.9 | 1.13 (0.82,1.55) | 1.12 (0.81,1.55) |
| DMPA any use | 10.4 | 8.7 | 1.20 (0.92,1.56) | 1.20 (0.92,1.56) |
| OCP any use | 21.5 | 21.4 | 0.99 (0.82,1.21) | 0.98 (0.81,1.20) |

CI: Confidence Interval, DMPA: depot-medroxyprogesterone acetate, ENG-IMP: etonogestrel 68 mg implants, LNG-IUS: levonorgestrel intrauterine system, OCP: combined oral contraceptive, OR: Odds Ratio.

[a] Models adjusted for weighted Rx-Risk score and menopausal hormone therapy use, and all medicine use types are included in the same model, with no use as the reference.

[b] Adjusted for obesity predictor probability score as a continuous variable (0-100), weighted Rx-Risk score and menopausal hormone therapy use, and all medicine use types are included in the same model, with no use as the reference.

[c] Obesity prediction score as a continuous variable. Score between 0 and 100 for probability of BMI ≥30 kg/m2.

[d] Breast cancer diagnosed at 50 years of age and older.

[e] Breast cancer diagnosed before 50 years of age.

Supplementary Table 12: Association between long-acting, progestin-based contraceptives and breast cancer risk with no contraception use as the reference (concessional sub-cohort)

| | Cases % | Controls % | Sensitivity analysis [a] OR (95% CI) |
|---|---|---|---|
| No use of any LARC/OCP | 80.4 | 83.2 | Reference |
| Mirena only | 2.4 | 1.8 | 1.42 (1.26,1.59) |
| Implanon only | 1.2 | 1.1 | 1.24 (1.06,1.46) |
| Depo only | 1.7 | 1.9 | 1.02 (0.89,1.17) |
| OCP only | 11.3 | 9.6 | 1.28 (1.21,1.36) |
| Use of more than one LARC/OCP | 3.0 | 2.4 | 1.38 (1.23,1.54) |

CI: Confidence Interval, DMPA: depot-medroxyprogesterone acetate, ENG-IMP: etonogestrel 68 mg implants, LARC: long-acting, progestin-based contraception; LNG-IUS: levonorgestrel intrauterine system, OCP: combined oral contraceptive, OR: Odds Ratio.

[a] Adjusted for menopausal hormone therapy use and Rx-Risk score.

**Supplementary Figure**

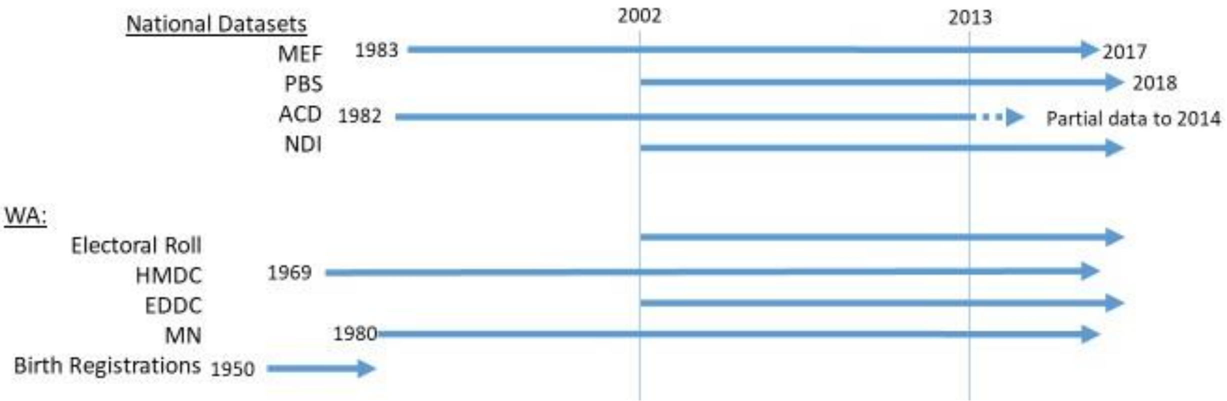


Supplementary Figure 1: Periods covered by the datasets.

ACD: Australian Cancer Database; EDDC: Emergency Department Data Collection; HMDC: Hospital Morbidity Data Collection; MEF: Medicare Enrolments File; MN: Midwives Notifications; NDI: National Death Index; PBS: Pharmaceutical Benefits Scheme.
